# Isolated p.H62L Mutation in the *CYP21A2* Gene in a Simple Virilizing 21-Hydroxylase Deficient Patient

**DOI:** 10.1155/2013/143781

**Published:** 2013-07-07

**Authors:** Melisa Taboas, Cecilia Fernández, Susana Belli, Noemi Buzzalino, Liliana Alba, Liliana Dain

**Affiliations:** ^1^Centro Nacional de Genètica Médica, ANLIS “Dr. Carlos G. Malbrán”, Buenos Aires, Argentina; ^2^Instituto de Biologìa y Medicina Experimetal, CONICET, Buenos Aires, Argentina; ^3^Divisiòn Endocrinología, Hospital Durand, Buenos Aires, Argentina

## Abstract

Congenital adrenal hyperplasia due to 21-hydroxylase deficiency accounts for 90%–95% of cases. This autosomal recessive disorder has a broad spectrum of clinical forms, ranging from severe or classical, which includes the salt-wasting and simple virilizing forms, to the mild late onset or nonclassical form. Most of the disease-causing mutations described are likely to be the consequence of nonhomologous recombination or gene conversion events between the active *CYP21A2* gene and its homologous CYP21A1P pseudogene. Nevertheless, an increasing number of naturally occurring mutations have been found. The change p.H62L is one of the most frequent rare mutations of the *CYP21A2* gene. It was suggested that the p.H62L represents a mild mutation that may be responsible for a more severe enzymatic impairment when presented with another mild mutation on the same allele. In this report, a 20-year-old woman carrying an isolated p.H62L mutation in compound heterozygosity with c.283-13A/C>G mutation is described. Although a mildly nonclassical phenotype was expected, clinical signs and hormonal profile of the patient are consistent with a more severe simple virilizing form of 21-hydroxylase deficiency. The study of genotype-phenotype correlation in additional patients would help in defining the role of p.H62L in disease manifestation.

## 1. Introduction

Congenital adrenal hyperplasia (CAH) due to 21-hydroxylase deficiency (OMIM 201910) accounts for 90%–95% of CAH cases [1, 2]. This autosomal recessive disorder, which is the most frequent inborn error of metabolism, has a broad spectrum of clinical forms, ranging from severe or classical, which includes the salt-wasting (SW) and simple virilizing (SV) forms, to the mild late onset or nonclassical form of CAH (NCCAH) [1]. 

The affected enzyme, P450C21, is encoded by the *CYP21A2* gene, located together with a 98% nucleotide sequence identity CYP21A1P pseudogene, on chromosome 6p21.3. Due to the high degree of identity between this gene and its pseudogene, most of the disease-causing mutations described are likely to be the consequence of nonhomologous recombination or gene conversion events [3, 4]. In addition, more than 130 rare point mutations that arise independently of the pseudogene and that were found specific to a population or a single family, have been described to date (for details visit http://www.hgmd.cf.ac.uk). Most of the patients are compound heterozygotes and their phenotype depends on the underlying combination of mutations they have [5].

The change p.H62L is one of the most frequent rare mutations of the *CYP21A2* gene. Based on patients' phenotypes and functional studies, it has been proposed that the p.H62L allele represents a mild mutation, which may be responsible for a more severe phenotype only when associated with another mild mutation on the same allele [6, 7]. Moreover, by *in silico* analyses using as a template the crystallized bovine CYP21 that shares 79% sequence identity with the human CYP21A2 protein, Haider et al. (2013) associate this mutation to the mild form of the disease [8]. 

Herein we describe a recently genotyped patient from our cohort carrying an isolated p.H62L mutation in one allele and the severe c.283-13A/C>G mutation in the other one. Although a mild NCCAH phenotype was expected, the patient presents a classical SV form of the disease. 

## 2. Case Report

### 2.1. Clinical and Hormonal Evaluations

Endocrine and genetic evaluations were conducted at the Division Endocrinología of the Hospital Durand and at the Centro Nacional de Genética Médica, Buenos Aires, Argentina. Patient was included following the diagnostic criteria already described [9, 10]. 17-Hydroxyprogesterone (17OHP), androstenedione (A_4_), and testosterone (T) were assayed by radioimmunoassay (RIA) using a commercial kit from Diagnostic System Laboratory (DSL), Houston, TX, USA. Plasma Renin Activity (PRA) was measured using a commercial kit from Renin, BioChem Immuno System, Rome, Italy (MAIA), as previously described [9, 10].

The patient, a 20 year-old woman, was referred from the paediatric unit for ongoing management of simple virilising form of congenital adrenal hyperplasia due to 21-hydroxylase deficiency. She presented genital ambiguity with almost complete labial fusion and clitoromegaly since birth; however medical attention was achieved at the age of 3.5 years. She presented a single urogenital orifice and a 2.4 cm clitoris with phallic appearance and no palpable gonads, advanced bone age of 5.3 years old, and 1.04 m height (P90–97). Plasmatic levels of 17 *α*-hydroxyprogesterone were >66 nmol/L (absolute values of the hormone in diluted sera were not available). She also presented increased plasma rennin activity of 9.5 ng/mL/h (normal values 0.15–2.33). She received oral hydrocortisone and 9*α*-fludrocortisone (0.05 mg per day). At age of 6, the patient underwent successful genital reconstruction with clitoral reduction and flap vaginoplasty. She had menarche at age 10.8 and regular menses since then. Due to adequate treatment compliance, she achieved a 1.55 m final height and had satisfactory sexual intercourse since the age of 19 years. When transferred to adult unit, treatment diagram was switched to prednisone 3 mg taken nightly. Given that basal plasma rennin activity was still increased (7.1 ng/mL/h), 9*α*- fludrocortisone treatment was kept. Under this treatment regimen, serum hormonal levels showed 17*α*-hydroxyprogesterone 3.3 nmol/mL, androstenedione 5.23 nmol/mL, and total testosterone 1.11 nmol/L.

### 2.2. Molecular Analyses

After written informed consent was obtained, DNA was isolated from peripheral blood leucocytes, and the 10 most frequent point mutations in the *CYP21A2* gene were screened, following methodology previously established in our laboratory [9, 10]. Briefly, *CYP21A2 *gene was specifically amplified by PCR in three overlapping fragments, and each mutation was screened by allele-specific PCR or PCR-RFLP in a second round of PCR using one of these previously amplified fragments as templates. Samples from patients who presented at least one nondetermined allele were further analyzed by direct sequencing covering the entire gene and proximal promoter regions of *CYP21A2* in four gene-specific overlapping fragments as previously described [11]. In addition, two previously described regulatory regions [12, 13] were also analyzed by direct sequencing. 

**Figure 1:**
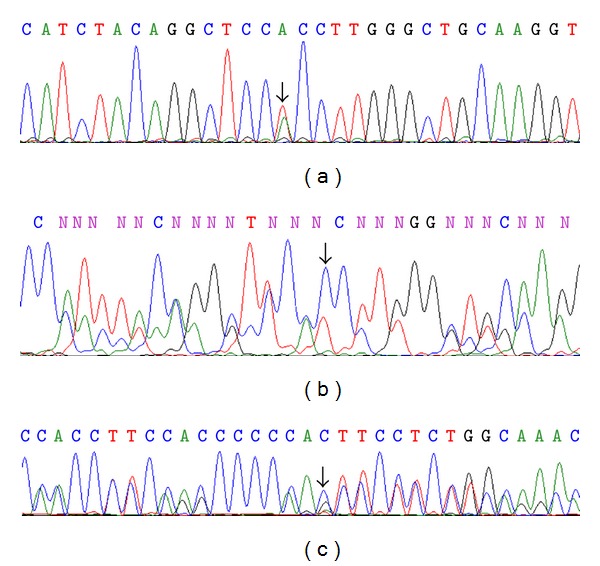
Representative electropherograms of the mutations found in the patient. The mutations are shown by arrows. (a) g.185A>T (p.H62L) mutation. (b) Reverse complement electropherogram showing the deduced presence of g.185A>T (p.H62L) on the allele carrying the g.562_568delG polymorphism. (c) IVS2-13 A/C>G mutation on the homologous allele.

The combined strategy described above revealed the presence of c.283-13A/C>G and g.185A>T (p.H62L) mutations, both in heterozygosis (Figures 1(a) and 1(c)). Even though the parents were not available, the presence of 6G or 7G at a polymorphic site in intron 2 (g.562_568delG) allowed the identification of both mutations localized in *trans *(Figure 1(b)). In addition, the patient presented 3 polymorphic variants in heterozygosis: g.692A>G (p.K102R) in exon 3, g.1130C>G (p.D183E) in exon 5, and g.2702A>G (p.N493S) in exon 10, thus further excluding a putative allele dropout commonly found in *CYP21A2* gene amplifications [14]. No variations were found in the regulatory regions analyzed. 

## 3. Discussion

 The adrenocortical 21-hydroxylase is one of the key enzymes in glucocorticoid and mineralocorticoid biosynthesis. Mutations in the *CYP21A2* gene have been reported in individuals affected with CAH due to 21-hydroxylase deficiency. To date, a great number of different mutations in the *CYP21A2* gene have been described. Because CAH is a recessive disorder and most of the 21-hydroxylase deficient patients are compound heterozygotes with different mutations in both alleles, their phenotype depends on the underlying combination of mutations they have [5]. Even though most patients carry CYP21A1P-derived mutations, an increasing number of naturally occurring mutations have been found in disease-causing alleles in the last years. 

The change p.H62L is one of the most frequent rare mutations of the *CYP21A2* gene. To date, thirty-one 21-hydroxylase deficient patients have been reported carrying the non-derived-pseudogene p.H62L mutation [6, 7, 15–19]. Most of them presented a second mutation on the same allele, while only twelve patients (7 of the Taiwanese population) from among all the reported in bibliography showed no additional mutations *in cis* [6, 15, 17, 18]. 

By* in silico* analyses, a recent report predicts that the substitution of histidine by hydrophobic leucine may disrupt hydrogen bonding between H62 and G35 to reduce, but not eliminate, enzyme activity [8]. While functional assays also suggested that p.H62L is a mild mutation, presence of another mutation on the same allele disclosed a synergistic effect in reducing enzyme activity [6, 7]. 

We herein report a classical patient who presents an isolated p.H62L in one allele and the c.283-13A/C>G mutations in the other one. Unlike the mild phenotype found in those patients previously described with the isolated p.H62L mutation in compound heterozygosity with a severe one, clinical manifestations and hormonal profile of the patient herein described are consistent with a SV form of the disease. More severe clinical manifestations of the disease were found, by contrast, in those patients carrying p.H62L with another mild mutation on the same allele [6, 7]. Though the entire gene and promoter regions were sequenced in the DNA sample from the patient from our cohort, no additional mutations were found. No variations were either found when we analyzed, by direct sequencing, two previously described regulatory regions. Nevertheless, it should be noted that the patient reported in 2009 in Japan, who presented an isolated p.H62L in one allele and the severe cluster E6 (p.I236 M, p.V237 E, p.M239 K) mutation in the other one, disclosed an intermediate phenotype. She presented clitoral size at the upper limit for normal and 17-OHP levels between the SV and NCCAH forms [17].

Discordances between genotype and phenotype in 21-hydroxylase deficiency have already been described in previous reports. Indeed, a recent work by New et al. [20] found a direct genotype-phenotype correlation in less than 50% of the genotypes studied. All these observations as a whole may suggest that despite mildly impaired enzymatic activity of the isolated p.H62L variable suggested by* in vitro* functional studies, presence of other genetic or environmental factors may contribute to modulate the activity of the enzyme* in vivo.*

The study of genotype-phenotype correlation in additional patients presenting this mutation would help in defining the role of this variable in disease manifestation. In the meantime, the possibility that p.H62L mutation by itself may be associated with a more severe clinical form of 21-hydroxylase deficiency should be considered.
